# Hypertension and Socioeconomic Status in South Central Uganda: A Population-Based Cohort Study

**DOI:** 10.5334/gh.1088

**Published:** 2022-01-13

**Authors:** Aishat Mustapha, Joseph Ssekasanvu, Ivy Chen, Mary Kathryn Grabowski, Robert Ssekubugu, Godfrey Kigozi, Steven J. Reynolds, Ronald H. Gray, Maria J. Wawer, Joseph Kagaayi, Larry W. Chang, Wendy S. Post

**Affiliations:** 1 Department of Medicine, Johns Hopkins University School of Medicine, Baltimore, MD, United States of America; 2 Rakai Health Sciences Program, Kalisizo, Uganda; 3 Department of Epidemiology, Johns Hopkins Bloomberg School of Public Health, Baltimore, MD, USA; 4 Department of Epidemiology, Mailman School of Public Health, Columbia University, New York, USA; 5 Department of Epidemiology and Biostatistics, School of Public Health, Makerere University, Kampala, Uganda; 6 Division of Intramural Research, National Institute for Allergy and Infectious Diseases, National Institutes of Health, Bethesda, MD, USA; 7 Division of Infectious Diseases, Department of Medicine, Johns Hopkins School of Medicine, Baltimore, MD, USA; 8 Division of Cardiology, Department of Medicine, Johns Hopkins School of Medicine, Baltimore, MD, USA

**Keywords:** Hypertension, Socioeconomic status, global cardiovascular disease, cardiovascular disease, Rakai

## Abstract

**Background:**

Limited studies exploring the impact of socioeconomic status (SES) on hypertension in Africa suggest a positive association between higher SES and hypertension. The economic development in sub-Saharan African countries has led to changes in SES and associated changes in lifestyle, diet, and physical activity, which may affect the relationship between hypertension and SES differently compared with higher income countries. This cross-sectional study from a large population-based cohort, the Rakai Community Cohort Study (RCCS), examines SES, hypertension prevalence, and associated risk factors in the rural Rakai Region in south-central Uganda.

**Methods:**

Adults aged 30–49 years residing in 41 RCCS fishing, trading, and agrarian communities, were surveyed with biometric data obtained between 2016 and 2018. The primary outcome was hypertension (systolic blood pressure (BP) ≥ 130 mmHg or diastolic BP ≥ 80 mmHg). Modified Poisson regression assessed the adjusted prevalence ratios (PR) of hypertension associated with SES; body mass index (BMI) was explored as a potential mediator.

**Results:**

Among 9,654 adults, 20.8% had hypertension (males 21.2%; females 20.4 %). Participants with hypertension were older (39.0 ± 6.0 vs. 37.8 ± 5.0; p < 0.001). Higher SES was associated with overweight or obese BMI categories (p < 0.001). In the multivariable model, hypertension was associated with the highest SES category (aPR 1.23; confidence interval 1.09–1.38; p = 0.001), older age, male sex, alcohol use, and living in fishing communities and inversely associated with smoking and positive HIV serostatus. When BMI was included in the model, there was no association between SES and hypertension (aPR 1.02; CI 0.90–1.15, p = 0.76).

**Conclusion:**

Hypertension is common in rural Uganda among individuals with higher SES and appears to be mediated by BMI. Targeted interventions could focus on lifestyle modification among highest-risk groups to optimize public health impact.

**Key Messages:**

**What is already known about this subject?**

- Hypertension is an important modifiable risk factor for cardiovascular disease.
- There are few large epidemiological studies that investigate the relationship between hypertension and socioeconomic status in low-income countries.

**What are the new findings?**

- Hypertension is common among adults in rural South–Central Uganda, particularly among those with higher socioeconomic status.
- BMI is a mediator of the relationship between hypertension and socioeconomic status.

**How might it impact on clinical practice in the foreseeable future?**

- These findings suggest that public health interventions and community efforts to prevent chronic cardiovascular disease and hypertension should focus on lifestyle modification by elucidating obesity risk perception and health risk awareness, particularly among those of higher socioeconomic status.

## Introduction

Non-communicable diseases (NCDs), including cardiovascular disease, are emerging causes of mortality and morbidity in sub-Saharan Africa [1]. Cardiometabolic risk factors, including hypertension, obesity and diabetes, are increasing in low-income countries [2]. Hypertension is a major modifiable risk factor for cardiovascular diseases such as ischemic heart disease, heart failure and stroke [3]. Globally, hypertension has been estimated to cause ~54% of stroke and 47% of ischemic heart disease. Several environmental, genetic, and hormonal factors including age, family history, obesity, physical activity, sodium intake, alcohol consumption and tobacco use predispose individuals to develop hypertension [4].

Another emerging factor is socioeconomic status (SES). There is a well-established relationship between lower SES and an increased risk for hypertension in high income countries [5]. In large epidemiology studies evaluating these relationships, the representation of low-income sub- sub-Saharan Saharan African countries remains poor. It is important to study the relationship between SES and hypertension in resource constrained settings in low-income sub-Saharan countries where risk factors may differ from high income countries.

A few population-based studies suggest that hypertension is prevalent in sub-Saharan Africa affecting between 10% to 50% of individuals depending on the population studied; however, the specific impact of SES on hypertension has not been well determined [6]. A systemic review performed in 2007 suggests a higher prevalence of hypertension in urban compared to rural communities [6]. There is some evidence to suggest that economic growth and globalization in sub-Saharan African countries due to changes in SES, with associated lifestyle changes, trends in nutrition, westernization of diet with consumption of highly processed food, weight gain, and decreased physical activity, may affect the relationship between hypertension and SES differently compared with high income countries [7]. The prevalence of overweight and obese individuals has been on the rise over the past three decades and remain on the rise in sub-Saharan African populations [8]. In 2014, ~1.9 billion adults aged 18 or older were believed to be overweight. Similarly in Africa, the prevalence of obesity has doubled between 1990 and 2014 from ~5.4 million to 10.6 million individuals [8]. We conducted a study to examine the relationship and mediators between SES and prevalence of hypertension in rural south-central Uganda.

## Materials and Methods

### Study Sample

The Rakai Community Cohort Study (RCCS) is an open population-based cohort of individuals aged 15 to 49 in rural agricultural, fishing, and trading communities. RCCS methods have been detailed previously [9]. Briefly, the RCCS conducts a household census with consenting residents to collect demographic, behavioral and health data. This cross-sectional study utilized RCCS data from the survey conducted between 2016 and 2018, which included 9654 individuals between ages 30 to 49, excluding 88 individuals without recorded SES data. We limited our study to these ages given that the risk of hypertension and other cardiovascular diseases are known to increase with age, and blood pressure was only collected on these age groups.

### Ethics Statement

Approval for the study was obtained from the Research and Ethics Committee of the Uganda Virus Research Institute (UVRI: GC/127/20/12/7), the Ugandan Council for Science and Technology and the Johns Hopkins School of Medicine Institutional Review Board (IRB00268735).

### Measurement of SES using Household Assets

Household asset-based measure (ABM) was used to assess SES as described elsewhere [10]. The RCCS census collected information on nine household assets including home construction (modern materials used for the roof, walls, and floor); access to a latrine; and electricity, and possessions of modern objects (car, motorcycle, bicycle, and radio). Principal component analysis (PCA) was used to identify and weigh assets that factor into SES [10]. The factor loadings represent the importance of each asset to SES. An ABM score was calculated for each household and standardized using Z-scores to define approximate quartiles of low, low- middle, high-middle, and highest SES. Only households in which there were no missing data for any of the nine asset variables were included in the analysis.

### Blood Pressure Measurement

All blood pressure measurements were performed by trained RCCS staff using a digital automatic blood pressure monitor (OMRON Model M6) with appropriate cuff sizes. Participants were asked to sit for at least 15 minutes with legs uncrossed. The cuff was placed above the elbow with lower band positioned 1–2 cm above the elbow joint with the elbow supported. The level of the cuff was kept at same level as the heart during measurement. Only one blood pressure reading was available for participants until halfway through the survey round after which two blood pressure measurements were obtained. Participants rested for at least three minutes in between blood pressure readings. We used the second blood pressure measurement in this latter situation. Hypertension was defined as a binary variable based on a systolic blood pressure (BP) ≥ 130 mmHg or diastolic BP ≥ 80 mmHg per the 2017 ACC/AHA guidelines [11]. We further stratified by the different stages of hypertension: stage 1 (≥130/80 mmHg), stage 2 (≥140/90 mmHg) and severe hypertension (≥180/120 mmHg). Information about BP medications was not available for every participant due to non-response.

### Body Mass Index

Weight in kilograms and height in cm were measured with a portable weighing scale and measuring rod (SECA scale) to determine body mass index (BMI). BMI classes were defined according to 1995 World Health Organization standards as underweight ≤ 18.5 kg/m^2^, normal 18.5–24.9 kg/m^2^, overweight as 25.0–29.9 kg/m^2^ and obese BMI as ≥30.0 kg/m^2^ [12].

### Smoking and Alcohol Use

Smoking and alcohol use were assessed through self-reported current use of any alcohol, cigarettes, tobacco, and pipe use and duration of use over weeks, months, and years.

### HIV Serostatus

Free HIV testing services were available at the time of interview with referral to combination HIV intervention as appropriate. HIV serostatus was determined using a validated three rapid HIV test algorithm. Blood samples were also taken for HIV confirmation.

### Community Type

Communities were categorized as agrarian, trading or fishing based on the major occupation of inhabitants.

### Statistical Analysis

Analyses were conducted using Stata Version 15.0 (STATA Corp, College Station, Texas, USA). Means and standard deviation (SD) were reported for continuous variables and percentage used for categorical variables. Two sample t tests were utilized to compare continuous variables and chi square test to compare categorical variables. Descriptive statistics and univariate analyses explored associations between key demographic and clinical characteristics by hypertension status. Multivariate analyses used modified Poisson regression with robust estimate of variance to assess the association between hypertension (dependent variable) and SES (predictor), adjusting for clinically relevant variables. Associations were expressed as prevalence ratios (PR) and 95% confidence intervals (CI). Models were adjusted for age, sex, current smoking, alcohol use, HIV serostatus, and community type. Models were performed with and without adjustment for BMI.

## Results

The baseline characteristics of participants aged 30–49 by hypertension status are summarized in ***Table 1***. Hypertension was present in 20.8% of study participants. Participants with hypertension were older than those without hypertension (39.0 ± 6 vs. 37.8 ± 5; p < 0.001), and more were in the overweight and obese BMI categories. Overall, 21% of individuals were overweight and only 9% of individuals were in the obese BMI category. Additionally, more hypertensive individuals resided in the fishing communities and reported alcohol use. Hypertension was less prevalent in people living with HIV and smokers. Severe hypertension was rare and only present in 1% of the cohort. Stage 2 hypertension was present in 17% of the cohort. Most participants had stage 1 hypertension. The severity of hypertension was related to advancing age and greater BMI.

**Table 1:** Baseline characteristics by hypertension status.


CHARACTERISTICS	HYPERTENSION							

	NO		YES		TOTAL	P-VALUE
		
	N.	%	N.	%	N.	

**Overall**	7579	79.2	1985	20.8	9654	

**Age (years)**						

**30–34**	2573	81.4	588	18.6	3161	**<0.001**

**35–39**	2268	82.8	471	17.2	2739	

**40–44**	1731	76.1	543	23.9	2274	

**45–49**	1007	72.5	383	27.5	1390	

**Socioeconomic status**						

**Low**	2284	79.7	582	20.3	2866	0.11

**Low – Middle**	1389	80.3	341	19.7	1730	

**High – Middle**	2042	79.6	522	20.4	2564	

**Highest**	1864	77.5	540	22.5	2404	

**SEX**						

**Male**	3535	78.8	949	21.2	4484	0.35

**Female**	4044	79.6	1036	20.4	5080	

**HIV SEROSTATUS**						

**Positive**	2118	81.6	478	18.4	2596	**0.001**

**Negative**	5439	78.4	1502	21.6	6941	

**ALCOHOL USE**						

**Yes**	4012	78.2	1122	21.8	5134	**0.004**

**No**	3567	80.6	863	19.4	4430	

**SMOKER**						

**Yes**	1035	82.2	221	17.8	1256	**0.003**

**No**	6544	78.8	1764	21.2	8308	

**OCCUPATION**						

**Agricultural/Housework**	3439	80.6	825	19.4	4264	**0.02**

**Bar\Restaurant**	322	76.1	101	23.9	423	

**Fishing**	732	79.1	194	20.9	926	

**Trade\Shopkeeper**	1317	77.5	381	22.5	1698	

**Other**	1769	78.5	484	21.5	2253	

**BMI**						

**Underweight**	538	84.1	96	15.9	634	**<0.001**

**Normal**	4985	82.4	1058	17.6	6043	

**Overweight**	1491	73.9	521	26.1	2012	

**Obese**	529	63.8	296	36.2	825	

**COMMUNITY TYPE**						

**Agrarian**	3429	81.5	780	18.5	4209	**<0.001**

**Fishing**	1889	75.6	611	24.4	2500	

**Trading**	2261	79.1	594	20.9	2855	


**Figure 1:**
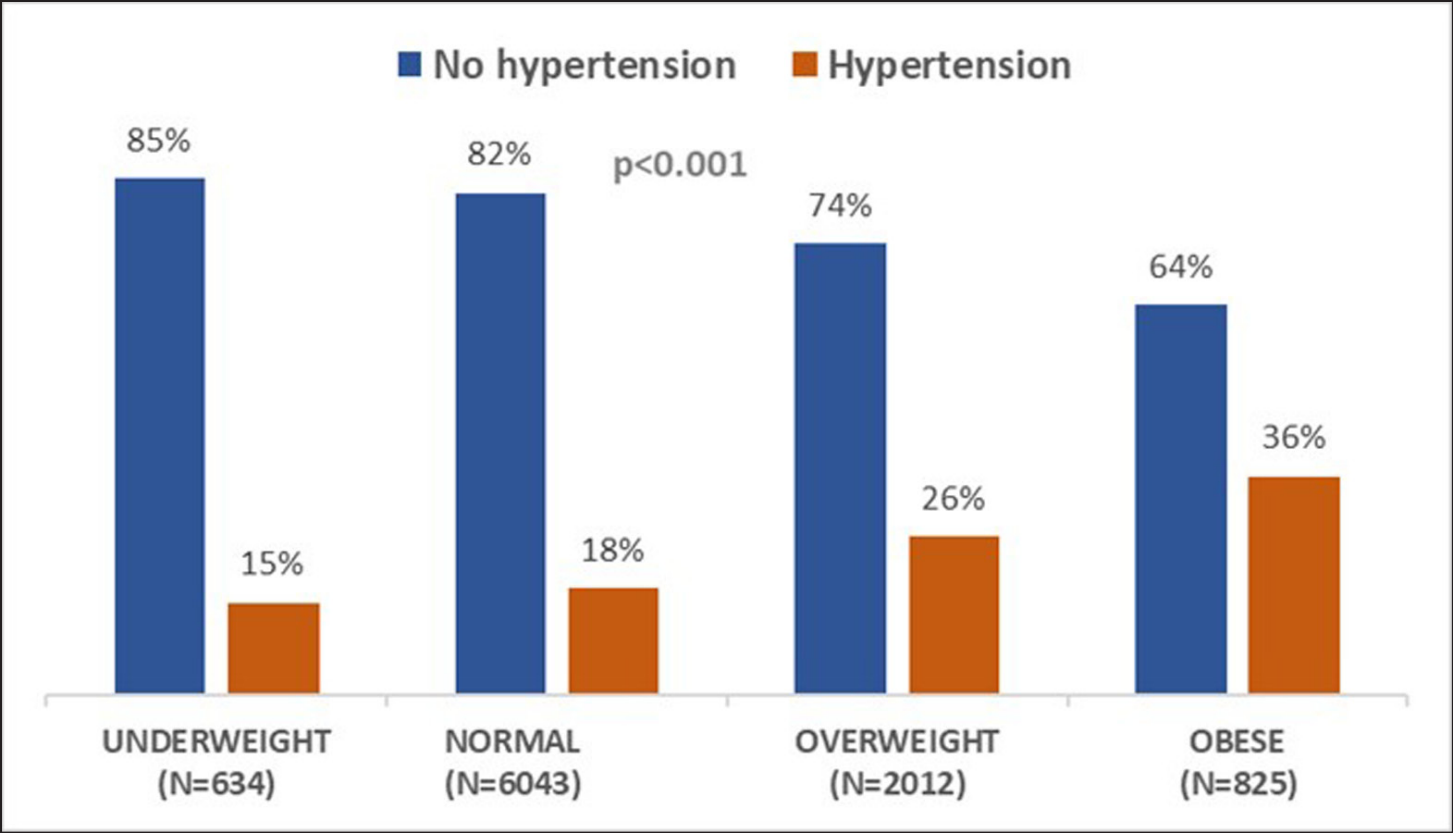
Prevalence of Hypertension by BMI Category.

**Figure 2:**
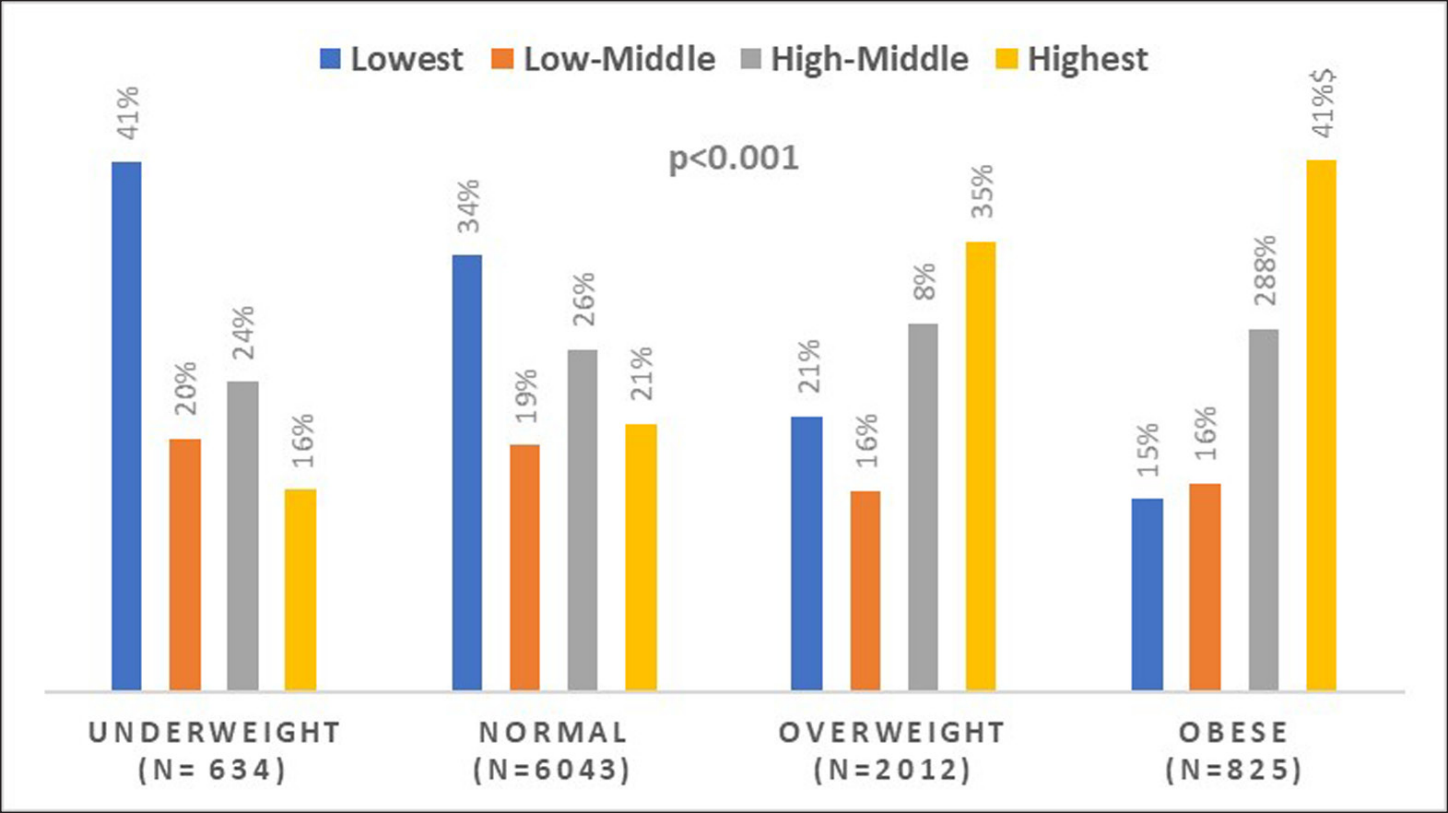
SES by BMI Category.

**Figure 3:**
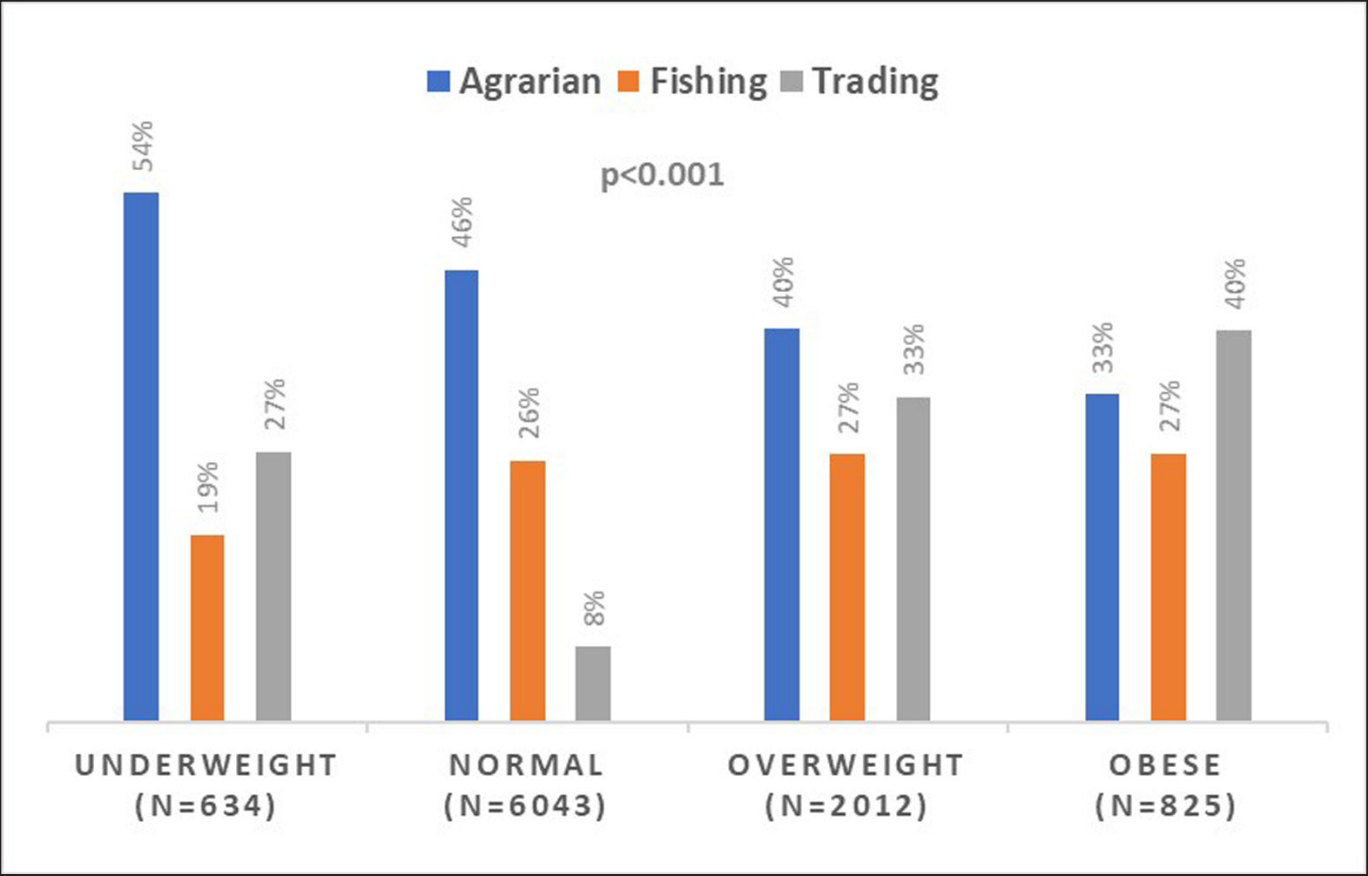
Community Type by BMI Category.

***Figure 1*** shows the unadjusted prevalence of hypertension by BMI categories. The prevalence of hypertension increased from underweight to obese BMI categories (15% vs. 36%; p < 0.001). ***Figure 2*** displays SES by BMI categories. There is a graded relationship between increasing SES category and increasing BMI. For example, 41% of individuals in the highest SES categories were obese compared to 16% in the lowest SES categories (p for trend < 0.001). Lastly, ***Figure 3*** demonstrates BMI categories by community type. Among individuals in the agrarian communities, 54% are underweight while a higher proportion of overweight and obese categories reside in fishing and trading communities.

Two multivariable models are presented in ***Table 2***. Both models are adjusted for age, sex, SES, current smoking, alcohol use, HIV serostatus, and community type. Model 1 is additionally adjusted for BMI, while Model 2 does not include BMI. In Model 1, there was a higher prevalence of hypertension among older individuals, males, residents of fishing community and individuals who drink alcohol. The risk of hypertension was lower among people living with HIV and smokers. Hypertension was not related to SES in Model 1, which was adjusted for BMI. This is consistent with the hypothesis that BMI could be a mediator of the relationship between hypertension and socioeconomic status. In Model 2, without adjustment for BMI, there is a significantly increased prevalence of hypertension among individuals in the highest SES category (PR 1.23, 95% CI 1.09–1.38) and in individuals residing in trading communities([PR] 1.12, 95% CI 1.01–1.24).

**Table 2:** Predictors of Hypertension from Multivariate Analysis Models with and without BMI. * Table reports RR of hypertension among variables depicted above from modified Poisson regression with robust variance. * Highlighted rows show differences between both models.


	MODEL 1: WITH BMI			MODEL 2: WITHOUT BMI		
	
VARIABLE	PREVALENCE RATIO (PR)	95% CI	P-VALUE	PREVALENCE RATIO	95% CI	P-VALUE

**Male Sex**	**1.22**	**1.12–1.34**	**<0.001**	0.99	0.91–1.07	0.86

**Community Type**						

**Agrarian**	**Reference**					

**Fishing**	**1.38**	**1.24–1.53**	**<0.001**	**1.62**	**1.46–1.80**	**< 0.001**

**Trading**	1.07	0.97–1.18	0.150	**1.12**	**1.01–1.24**	**0.020**

**Asset Based Measure (SES)**						

**Low**	**Reference**					

**Low – Middle**	0.97	0.86–1.10	0.61	1.04	0.92–1.18	0.51

**High – Middle**	0.99	0.89–1.10	0.88	1.11	0.99–1.24	0.079

**Highest**	1.02	0.90–1.15	0.76	**1.23**	**1.09–1.38**	**0.001**

**Smoker (yes)**	**0.78**	**0.68–0.89**	**<0.001**	**0.74**	**0.65–0.84**	**< 0.001**

**Age (years)**	**1.03**	**1.02–1.04**	**<0.001**	**1.03**	**1.02–1.04**	**< 0.001**

**Body Mass Index (BMI)**	**1.06**	**1.05–1.07**	**<0.001**			

**Alcohol Use (yes)**	**1.12**	**1.03–1.21**	**0.006**	**1.14**	**1.05–1.23**	**0.002**

**HIV Seropositive**	**0.85**	**0.77–0.93**	**0.001**	**0.79**	**0.71–0.87**	**< 0.001**

**Number of observations (N) = 9487** **Akaike’s Information Criterion (AIC): 9861** **Bayesian Information Criterion (BIC): 9946**				**Number of observations (N) = 9537** **Akaike’s Information Criterion (AIC): 10057** **Bayesian Information Criterion (BIC): 10136**		


## Discussion

Hypertension is an easy clinical diagnosis yet is frequently referred to as the silent killer, as individuals may remain asymptomatic even when hypertension is severe. Blood pressure management is crucial to reducing the risk of cardiovascular disease. This analysis in the rural Rakai region of South-Central Uganda shows that hypertension is positively associated with male sex, older age, alcohol use and living in fishing communities, and negatively associated with smoking and HIV positive serostatus.

In the model unadjusted for BMI, there was a strong association between hypertension and the highest SES category. In the model adjusted for BMI, there was no longer an association between SES. This shows that BMI may be a mediator of the relationship between hypertension and SES.

The increase in prevalence of hypertension and obesity among other cardiometabolic risk factors in sub-Saharan Africa occurs in parallel to economic transition and lifestyle changes. Western diets and fast-food restaurant style eating have become increasingly common due to globalization of food markets and fast-food chains [2]. This leads to consumption of calorically dense foods without compensating with increase in physical activity [2]. Another possible explanation is that the availability of westernized foods might be dictated by price in many developing countries including Uganda and thus access to these foods is affected by socioeconomic status. If westernized foods are more expensive and less available to street vendors then they become less available to individuals of lower SES [13]. This phenomena may correspond with the rising prevalence of obesity among higher SES individuals in sub-Saharan Africa.

Prior studies of the association between hypertension and SES show conflicting findings. A large meta-analysis evaluated the prevalence of hypertension in 16 high income and 16 middle- and low-income countries in three regions. This showed an increased risk of hypertension among lower SES individuals in high income regions, but an inverse association in three African countries [14]. Furthermore, a cross-sectional survey focused specifically on urban clinics in 12 sub-Saharan countries found that low wealth was associated with uncontrolled hypertension [15]. However, this study focused on urban centers with different population and community makeup in comparison to our study which focused on rural regions in Rakai district. The idea that the relationship between hypertension and SES may differ in rural and urban populations in Sub-Saharan Africa has not been well studied.

Further cultural context is relevant to the association between hypertension with alcohol use among fishing community residents. Multiple studies show positive correlations between alcohol use and hypertension [16]. Uganda has one of the highest rates of alcohol use among sub-Saharan Africa with approximately 23.7 liters of alcohol consumed annually per capita [17]. In 2019, Uganda banned the consumption and sale of alcohol (45% proof) in small plastic packets due to concern for increasing alcohol abuse. Previous studies in Rakai found that fishing communities and employment as fishermen or bar/restaurant keepers were associated with higher alcohol consumption [18]. Our study further links alcohol to increased risk of hypertension in fishing communities.

There was an inverse relationship between hypertension and smoking observed in this study. This relationship also persisted with adjustment for BMI in Model 2 and is contrary to prior research showing an increased risk of hypertension with tobacco smoking. Notably, the questionnaire used in this study inquired about smoking of cigarettes, tobacco, or pipes but the proportion of smokers for each of these methods is unknown. However, products utilized span a wide range of smoked and smokeless tobacco products. Common smoked products were hand rolled cigarettes, pipes and shisha and smokeless products included stuff and chewed tobacco [19]. The relationship between hypertension and non-nicotine tobacco products is still unclear. Further analysis pertaining to the inverse relationship between hypertension and smoking as well as HIV positive serostatus will be addressed in future studies [20].

The results of this study could be used to tailor resources and interventions towards high-risk individuals and communities. It noteworthy that participants in our study are relatively young (ages of 30–49) and it is expected that the risk of hypertension and other cardiovascular diseases will increase with age. Hypertension control efforts could focus on individuals with the highest risk characteristics (i.e. males, alcohol users, residents of fishing villages, individuals of older age, and among the highest SES categories) if resources are limited to maximize impact. Given the association of hypertension with BMI, interventions could focus on education about lifestyle practices that decrease the risk of obesity. Structural interventions may also be needed.

## Strengths and Limitations

This study has several limitations. It is a cross-sectional study. Only one blood pressure measurement was used to determine hypertension during half the survey round. When two blood pressure measurements were recorded, we used the second blood pressure to determine hypertension. Additionally, there is likely an element of recall bias that can contribute to the collection of variables such as smoking and alcohol use. While we assessed the association between hypertension and behavioral risk factors such as alcohol and smoking, we were unable to study other important risk factors including BP medications, sodium(salt) intake, fruit and vegetable intake and physical activity that could further elucidate the role of BMI as a mediator of the relationship between hypertension and SES. Despite these limitations, the significant strengths of this study include its large sample size and the use of a standardized measurement for SES and other variables included in the study.

## Conclusion

This study identified high risk populations who may be good candidates for targeted hypertension interventions. The goal to decrease the prevalence of hypertension is a challenging global health priority, and preventive strategies could be tailored to specific populations to attain substantial public health impact in the setting of limited resources. Pragmatic, targeted evidence-based interventions are needed to increase awareness, diagnosis, and treatment of hypertension throughout sub-Saharan Africa.
